# Surgical Outcomes in Stage IV Pancreatic Cancer with Liver Metastasis Current Evidence and Future Directions: A Systematic Review and Meta-Analysis of Surgical Resection

**DOI:** 10.3390/cancers17040688

**Published:** 2025-02-18

**Authors:** Noah Clements, Jeremy Gaskins, Robert C. G. Martin

**Affiliations:** 1 The Hiram C. Polk, Jr., MD Department of Surgery, Division of Surgical Oncology, University of Louisville School of Medicine, Louisville, KY 40292, USA; naclem01@louisville.edu; 2 The Department of Bioinformatics and Biostatistics, University of Louisville School of Medicine, Louisville, KY 40292, USA; jeremy.gaskins@louisville.edu

**Keywords:** surgical selection factors, patient selection, oligometastatic, pancreatic cancer, pancreaticoduodenectomy, survival, pancreatectomy, hepatic metastasis

## Abstract

Background/Objectives: There is increasing evidence that a subset of patients with stage IV pancreatic ductal adenocarcinoma (PDAC) and liver-only metastasis may benefit from surgical resection of both the primary tumor and metastatic lesions. Methods: A meta-analysis and systematic review were conducted in patients with stage IV PDAC and hepatic-only metastasis. A comprehensive literature search (January 2015–June 2023) was performed using PubMed with keywords including “pancreatic cancer”, “oligometastatic”, and “surgery”. Results: Sixteen articles were included in the final review and characterized based on patient selection factors and prognostic indicators. Seven studies reported hazard ratios (HRs) or Kaplan–Meier curves for survival in synchronous resected cohorts versus chemotherapy/palliation alone, which indicated a statistically significant survival benefit in the resection cohorts (pooled HR: 0.41, 95% CI: 0.31–0.53, *p* < 0.01). Prognostic indicators for synchronous and metachronous resection included lower pre-operative CA19-9, negative margin status of the primary tumor, moderate-to-well-differentiated tumors (grades I–II), and receiving pre-operative chemotherapy. Conclusions: Surgical/ablation selection factors are evolving, with priorities on (1) response to induction chemotherapy, (2) ability to achieve R0 resection, and (3) minimally invasive approaches remaining critical to optimal patient selection. Standardized radiologic and tumor marker evaluation and response to neoadjuvant therapy and optimizing performance status are critical to improved outcomes.

## Simple Summary

Currently, the standard of care for patients diagnosed with metastatic pancreatic adenocarcinoma involves chemotherapy without surgical evaluation or treatment. However, increasing evidence suggests that a subset of these patients may benefit from resection/ablation. There remains a lack of standardization regarding the selection of patients who would derive the most benefit from surgical intervention. This review aims to summarize the current literature on hepatic metastatic pancreatic adenocarcinoma patients who may benefit from surgery. Furthermore, it explores patient factors associated with improved survival following surgical intervention for hepatic metastatic pancreatic adenocarcinoma.

## 1. Introduction

Pancreatic ductal adenocarcinoma (PDAC) remains a devastating malignancy and was the fourth most common cause of cancer-related death in the United States in the year 2020 [1,2]. In recent years (2001–2018), studies have indicated a rising incidence of PDAC in younger populations without definite known cause [3,4]. Pancreatic cancer is projected to become the second most common cause of cancer-related deaths in the United States by the year 2030 [5].

Historically, pancreatic cancer has been associated with poor survival (<10%) over five years [1,6]. Advancements in multimodal chemotherapy, improved peri-operative patient care, high surgical expertise, and innovative surgical techniques have collectively led to enhanced survival rates for localized PDAC (Stages I, II, and III). In certain subgroups eligible for curative-intent resection, the 5-year survival has risen to approximately 40% or higher [7]. While chemotherapy is indicated in all stages of PDAC, resectability remains the major limiting factor of survival with only approximately 10–20% of PDAC being resectable at diagnosis under current guidelines [8]. A significant factor contributing to such low resection rates is the discovery of synchronous distant metastasis or vital vessel invasion (Stage III—Locally Advanced) at diagnoses in over 75% of adults in the United States [9]. Surgical treatment in resectable or borderline resectable disease is linked to the highest survival rates in PDAC patients when compared to systemic chemotherapy regimens alone [10]. However, for those with metastatic PDAC (mPDAC), systemic chemotherapy remains the sole recommended treatment [6].

Recent clinical trials have established two multi-chemotherapy regimens, gemcitabine/nab-paclitaxel (GnP) and FOLFIRINOX (FFX), as showing improved survival outcomes in patients with mPDAC. These regimens have now been adopted as the new standards of care [7,11,12]. Nevertheless, patients diagnosed with stage IV disease exhibit a notably low survival rate, with just 3.2% surviving over a span of five years [13]. In contrast to other cancers, notably colorectal, there is a lack of evidence regarding surgical ablation/resection of mPDAC [14]. The most common site of mPDAC is the liver [15]. Currently, studies indicate that the standard of care in colorectal cancer patients with hepatic metastasis is surgical treatment of both the primary and metastases with a 10-year survival of approximately 24% in select patients. In contrast, a consensus on the survival benefit and factors indicating increased survival post-resection in stage IV PDAC involving the liver has not been reached [16].

In recent years, there has been a rise in studies examining the role of curative-intent surgical resection for primary PDAC and liver metastatic lesions, often combined with systemic chemotherapy and/or non-thermal or thermal ablation, primarily for hepatic metastases, though select cases have utilized non-thermal ablation for the primary tumor. This surgical/ablative approach in cases of presumed oligometastatic disease is defined by a limited burden of metastasis to a single organ [14]. Oligometastasis is a term originally proposed by Hellman et al. that suggests there is a state between localized cancer and poly-organ metastasis in which the disease remains limited [17]. A recent retrospective analysis of six European centers found a survival benefit in oligometastatic hepatic PDAC patients who underwent synchronous resection of the primary and hepatic lesions to be associated with an increased survival over an exploration-only control group [18]. Currently, there is a lack of systematic reviews exploring the survival benefit of stage IV PDAC with liver-only oligometastasis who undergo surgical treatment compared to systemic chemotherapy or palliation alone [1,14]. There is also a lack of a consensus regarding selection factors for surgical candidacy and clinicopathological factors that may confer increased post-resection survival [19]. This systematic review and meta-analysis aimed to evaluate the survival benefit of surgery versus chemotherapy alone, surgical selection criteria, and prognostic indicators of survival for patients with stage IV PDAC and liver-only oligometastasis who underwent resection of both the primary and metastatic lesions.

## 2. Methods

### 2.1. Search Strategy and Exclusion Criteria

A literature search was performed in a comprehensive manner according to the Preferred Reporting Items for Systematic Reviews and Meta-Analyses guidelines (PRISMA guidelines) [20]. An electronic search of PubMed and EBSCO databases was performed using the combinations of ‘pancreatic cancer’, ‘oligometastatic’, and ‘surgery’ in the search fields. The search was limited to recent studies published between January 2015 and June 2023. All abstracts were comprehensively reviewed by two examiners for potential inclusion in the review. Criteria for inclusion were papers that investigated prognostic factors and patient outcomes following resection of stage IV pancreatic adenocarcinoma and liver oligometastatic disease. Articles were excluded based upon the following criteria: non-English language, conference notes, and case reports. Additionally, articles were excluded if they did not report overall survival data for patients who underwent resection. After removing duplicates, the full text of the remaining articles was extracted and examined with regard to the outcomes of interest. Texts that did not include adequate data or had non-specific results regarding outcomes following stage IV pancreatic cancer resection were excluded from the final cohort of studies. If significant cohort overlap was suspected, the most recent study was included in the review. Due to scarcity of manuscripts assessing the primary outcomes, studies that reported cohorts with primarily hepatic oligometastasis but still included patients with peritoneal or lymphatic oligometastasis were included if less than a third of the cohort had non-hepatic metastasis. Additionally, studies that included other malignant entities of the pancreas including acinar cell carcinoma, neuroendocrine tumors, and cholangiocarcinoma were excluded from this review. This review was performed in accordance with the PRISMA (Preferred Reporting Items for Systematic Reviews and Meta-Analyses) guidelines and has not been registered.

### 2.2. Definitions

Currently, there is a lack of consensus on a standardized definition of oligometastasis in pancreatic cancer, particularly concerning the number and size of metastases [14]. Nevertheless, there is emerging consensus that having three or fewer liver lesions is a threshold that is starting to gain acceptance [14]. This review deliberately adopted a broad interpretation of PDAC with resectable hepatic metastasis, as outlined by the authors, in order to obtain an improved census of current practices. Pre-operative treatment was defined as any chemotherapy, radiation for primary PDAC or hepatic metastases, or thermal ablation (MWA/RFA/Cryo) for hepatic metastases. Overall survival (OS), disease-free survival (DFS), mortality, morbidity, and major morbidity were reported as defined in each study. Patient selection factors were operationally defined as any discernible criteria employed within the study to delineate eligibility for surgical intervention and were extracted when reported. Timing of metastasectomy surgery was divided into two categories. Synchronous resection was defined as the simultaneous removal of the primary tumor and metastases. Metachronous resection involved performing two or more separate operations at different time points to remove the primary tumor and the metastatic lesions. Indicators of overall survival were defined as any factors reported by the authors that afforded a statistical survival benefit in primarily the resection cohort. Timing of metastasis was also divided into two definitions. Synchronous metastasis was defined as metastatic lesions having been diagnosed at the same time or within six months of the primary tumor. Metachronous metastasis was defined as having diagnosed metastatic lesions beyond six months from the primary tumor diagnosis.

### 2.3. Quality Assessment

A scoring system adapted from the methodological index for non-randomized studies (MINORS) [21] was implemented to generate a study score. For non-randomized studies, points were awarded for eight different categories. Zero points for a category not being reported, one point for reported but inadequate, and two points for reported and adequate. The eight categories include a clearly stated aim, inclusion of consecutive patients, prospective collection of data, endpoints appropriate to the aim of the study, unbiased assessment of the study endpoint, follow-up period appropriate to the aim of the study, loss to follow up less than 5%, and prospective calculation of the study size. An additional four categories are added for a comparative study. These include an adequate control group, contemporary groups, baseline equivalence of groups, and adequate statistical analysis.

### 2.4. Outcomes of Interest and Data Extraction

The primary outcomes examined in this review encompassed the assessment of overall survival (OS) subsequent to the resection of primary and metastatic lesions, along with the identification of prognostic factors for prolonged post-operative survival and surgical selection criteria. Median overall survival (OS) and their confidence intervals were obtained when available for the resection cohorts and non-resection cohorts. In addition to median OS, hazard ratios comparing survival in resected versus non-resected cohorts were extracted. Secondary outcomes included disease-free survival (DFS). Median months of DFS and their confidence intervals were extracted when reported. Prognostic indicators of survival for resection cohorts were extracted when reported. Clinicopathological data were obtained for the resection cohort in each study, which included type of operation for both the primary PDAC and hepatic metastasis, timing of resection (metachronous vs. synchronous), post-operative complications, mortality, morbidity, pre-operative, and adjuvant chemotherapy regimens. Furthermore, data on pre-operative imaging, biochemical, and radiological tumor response were extracted when available. Patient selection factors included radiologic response to pre-operative therapy as defined by the author, tumor markers (CEA and CA19-9), classification of oligometastasis, type of pancreatic resection, differentiation of primary/metastatic lesions, resection margin status, and surgeon discretion.

### 2.5. Statistical Analyses

Meta-analysis was performed for the results of included studies when applicable. For comparison of OS in resected cohorts versus chemotherapy and non-surgical treatment cohorts, hazard ratios and 95% confidence intervals were extracted if available. Due to the degree of heterogeneity in multivariate analyses across studies, only univariate hazard ratios were extracted for meta-analysis. In addition to hazard ratios, Kaplan–Meier curves that included number at risk were extracted for meta-analysis. The hazard ratio and standard error were computed using method eleven [22]. Meta-analysis across these hazard ratios was performed using the random effects model on log-HR to account for expected heterogeneity. Analysis was performed using R statistical software, version 4.3.3 with the dmetar package [23].

## 3. Results

### 3.1. Literature Selection

**Figure 1:**
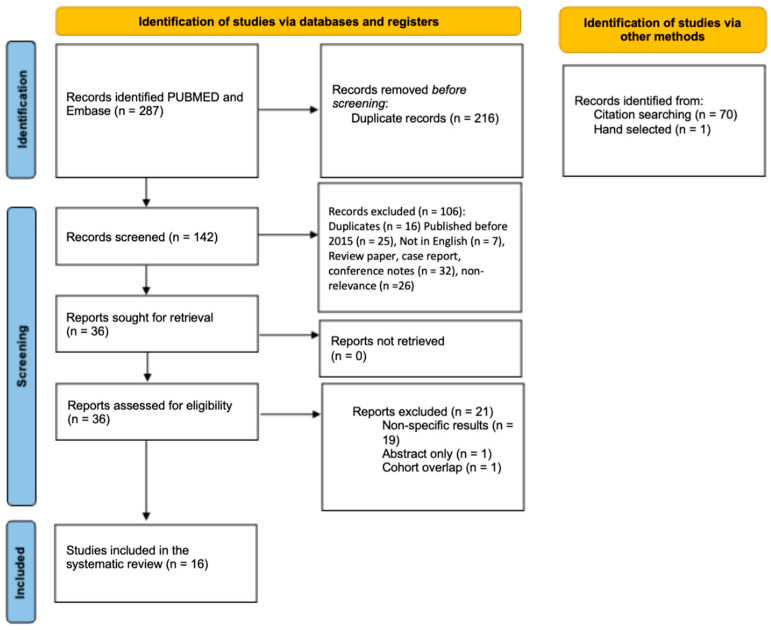
PRISMA diagram of systematic review.

The initial search yielded 287 results, of which 216 duplicates were removed prior to screening (Figure 1). An additional 71 studies were identified through citation searching and hand selection. From the combined 142 records, 106 additional records were excluded during screening based upon publication date prior to 2015, duplications, non-English language, review paper, case reports, conference notes, and not assessing the primary outcomes. The remaining 36 studies were retrieved, and the full text was examined for each. Upon review, 21 more results were excluded, which resulted in 16 studies for the final review [18,24,25,26,27,28,29,30,31,32,33,34,35,36,37,38].

### 3.2. Study Characteristics

The final studies included in the review were published from 2016 to 2023 (Table 1). The majority of studies [24,25,26,27,28,29,30,31,32,33,34,35,36,38] were retrospectively completed at single- or multi-center institutions while two [18,37] were completed using a national registry database. Outcome data were only obtained for selected patients with stage IV PDAC and liver metastasis who underwent resection of the primary and the metastatic sites or patients who underwent resection of the primary tumor after complete or partial regression of hepatic metastasis following neoadjuvant chemotherapy. Outcome data involving metastatic sites other than the liver were not included unless the majority of the cohort had primarily liver metastasis while a subset had extra-hepatic metastasis. For all included studies, the strength score, country, and study period are also summarized in Table 1.

### 3.3. Surgical Procedures and Their Morbidity and Mortality

The majority of studies included in this review focused on resection of primary PDAC with hepatic metastasectomy (Table 2). In the selected studies, a combined 999 patients with stage IV PDAC underwent pancreatic resection with or without additional hepatic metastasectomy. A total of thirteen studies [18,24,25,26,27,29,30,31,32,33,35,36,38] reported 637 patients who underwent synchronous resection of the PDAC and hepatic metastatic sites. Three studies [27,28,35] reported 48 patients who underwent primary PDAC resection with metachronous resection of hepatic metastasis. Pausch et al. [37] reported 259 patients with PDAC and liver metastasis who underwent cancer-directed surgery; however, due to database restrictions, the authors were unable to report the timing of metastasis surgery. Crippa et al. [25] reported a split cohort, in which there was a complete or major radiologic response of hepatic metastasis following neoadjuvant treatment in a subset of patients. Of the 11 patients reported in Crippa et al., 3/11 (27%) patients had one liver metastatic lesion confirmed intra-operatively and underwent synchronous resection. The remaining 8/11 (73%) patients had no identifiable liver metastasis at re-staging and underwent primary PDAC resection. Takeda et al. [30] reported 5/10 (50%) patients with intra-operatively identifiable metastatic sites who underwent synchronous resection while the remainder underwent only resection of the primary PDAC. Frigerio et al. [34] reported a cohort of 52 patients who had complete radiologic response of the liver metastasis following chemotherapy and underwent resection of the primary PDAC only. Lu et al. and Bachellier et al. additionally reported 34 patients who underwent radiofrequency ablation (RFA) of the hepatic metastasis prior to resection of the primary PDAC. Shao et al. and Bachellier et al. reported 16 patients who underwent a combination of RFA and resection of hepatic metastasis in addition to the primary PDAC resection. Hong et al. reported a mixed cohort, of which 4/7 (57%) patients had liver metastasis. Among these patients, one underwent synchronous resection of the primary PDAC and liver metastasis with irreversible electroporation (IRE) (liver metastasis), one underwent liver resection followed with IRE in situ for the primary pancreatic lesion, one underwent left lateral segmentectomy with right liver ablation followed with IRE in situ, and one received 3D conformal radiation therapy for liver metastasis along with IRE in situ for the primary pancreatic lesion.

Thirteen studies [24,25,26,27,28,29,30,32,33,34,35,36,38] reported a combined 584 patients, for whom data on specific procedure type were able to be extracted. The operation of highest frequency for the primary PDAC was pancreaticoduodenectomy (PD) (n = 307/584). Other operations performed for the primary PDAC included distal pancreatectomy (DP) (n = 146/584), distal splenopancreatectomy (DSP) (n = 63/584), subtotal pancreatectomy (SP) (n = 2/584), IRE in situ (n = 5/584), distal pancreatectomy with celiac artery resection (DP-CAR) (n = 3/584), subtotal stomach-preserving pancreaticoduodenectomy (SSPPD) (n = 6/584), pylorus-preserving pancreaticoduodenectomy (PPPD) (n = 12/584), total pancreatectomy (TP) (n = 39/584), and total pancreatectomy with celiac artery resection (TP-CAR) (n = 1/584). Five studies [25,26,27,33,38] reported a combined 181 patients that contained data on the specific type of hepatic metastasis surgery that was performed. The operation of highest frequency for hepatic metastasis was atypical liver resection (n = 132/181). Other operations included segmentectomy (n = 18/181), hemi-hepatectomy (n = 9/181), wedge resection (n = 21/181), and unspecified liver resection (n = 1/181).

Seven studies [26,27,29,30,33,34,35] reported data on 30-day mortality that were able to be extracted. Six studies [26,30,33,34,35,38] reported no 30-day mortality among their cohorts (Table 2). Hackert et al. reported two patients that experienced 30-day mortality with one patient in the synchronous resection group (n = 1/62) and the other in the metachronous resection group (n = 1/23). Tachezy et al. reported one patient who underwent synchronous resection that experienced 30-day mortality (n = 1/69). Three studies reported data on 90-day mortality (range 0–5%). Ten studies reported data on post-operative complications that were extracted. Classification of complications varied across studies; however, the majority used the Clavien–Dindo grading system (CDC) [29]. Overall morbidity was reported by two studies (range 45–57.7%). Among six studies that reported data on specific types of post-operative complications, post-operative pancreatic fistula (grades A–C) was the complication of highest frequency (n = 63/251, 25%). Four studies reported major morbidity defined as CDC grades III–IV (range 17–37%).

### 3.4. Chemotherapy Regimens

Table 3 outlines twelve studies that reported data on adjuvant and neoadjuvant chemotherapy regimens. Nine studies reported a quantification of the types of neoadjuvant chemotherapy received by each patient. The nine studies reported a combined cohort of 408 patients, of which 66.2% (n = 270/408) underwent neoadjuvant chemotherapy. In the subset of patients that underwent chemotherapy, FOLFIRINOX (FFX) was the most frequently reported regimen (n = 168/270, 62.2%) followed by gemcitabine plus nab-paclitaxel (GnP) (n = 33/270, 12.2%). Shao et al. reported that 82% (n = 41/50) of the patients in their study received neoadjuvant therapy with either FFX or gemcitabine with or without nab-paclitaxel. Crippa et al. reported FFX or PEFG (cisplatin, epirubicin, 5-fluorouracil, gemcitabine)/PEXG (cisplatin, epirubicin, capecitabine, gemcitabine)/PDXG (cisplatin, capecitabine, docetaxel, gemcitabine)-based regimens used in 77% (n = 35/45) of their cohort; however, it is not clear which regimens were used in the subset that underwent resection (n = 11/45). Five studies reported data regarding the number of cycles of neoadjuvant chemotherapy (range 1–30 cycles).

Eleven studies reported data on adjuvant chemotherapy. Gemcitabine (GEM) was the most frequently used adjuvant therapy in three of the studies. Lu et al. reported an adjuvant regimen that was the same as the neoadjuvant regimen (FFX: n = 10/15, GnP: n = 5/15) for eight weeks followed by oral S-1 for 3–4 months (n = 11/15) or oral S-1 for 4–6 months (n = 4/15). Additionally, Takeda et al. reported an adjuvant regimen of oral S-1 for a duration of six months. Yang et al. reported 79.2% (38/48) of their cohort receiving various GEM-based adjuvant chemotherapies; however, these data included oligometastatic (n = 23/48) and non-oligometastatic patients (n = 25/48). Bachellier et al. reported 84.8% (n = 78/92) of patients receiving adjuvant chemotherapy without specifying the type of regimen utilized. Shao et al. reported that a combination of adjuvant chemotherapy, RFA, and radiotherapy was utilized but does not specify type of regimen. Hong et al. utilized adjuvant therapy in 57.1% (n = 4/7) of their cohort with FOLFOX being used in half. Two studies reported that adjuvant chemotherapy was used in a subset of patients but did not report frequency or type of regimen.

### 3.5. Surgical Selection Factors for Synchronous Metastasis

Table 4 outlines the criteria used by each study consisting of solely synchronous metastasis patients for surgical selection. Twelve studies reported data regarding surgical selection in synchronous metastatic patients [18,24,25,26,29,30,31,32,33,34,36]. The total cohort (n = 609) predominantly consisted of patients with synchronous hepatic-only metastatic PDAC. Two studies, Hank et al. and Hong et al., included mixed cohorts: Hank et al. reported hepatic-only metastases (n = 67/93) alongside patients with lymphatic or peritoneal metastases (n = 26/93), while Hong et al. included cases with hepatic metastases with or without peritoneal involvement (n = 4/7) and peritoneal/omental metastases only (n = 3/7), respectively.

The majority of patients in this cohort also received neoadjuvant therapy (Table 3). Regarding number of metastatic liver lesions as a criterion, six studies [18,24,26,29,32,36] did not set a maximum number of hepatic lesions for surgical selection. There were three studies [30,33,38] that required oligometastatic patients to have three or fewer hepatic lesions to be selected for surgical resection. Safi et al. required that patients have no more than four lesions to be selected for resection. Both Crippa et al. and Frigerio et al. mandated that patients either have zero to one lesion or complete regression, respectively, following neoadjuvant therapy. Regarding size of metastasis, only one study, Bachellier et al., explicitly stated that they considered surgery to a greater extent in patients with liver lesions no greater than three centimeters in size. All other studies did not explicitly state selection criteria with regard to size of liver lesions. Regarding liver locale of metastatic lesions, two studies employed criteria that were utilized for surgical selection. Safi et al. only considered surgery if the hepatic lesions were confined to a single liver lobe. Bachellier et al. selected patients for resection if their metastasis was confined to the sub-capsular liver space.

Not a single study utilized initial biomarker levels prior to neoadjuvant therapy as a surgical selection criterion. Seven studies utilized biomarker level response to neoadjuvant therapy as a selection criterion for surgery. Two studies, Crippa et al. and Bachellier et al., required specific reductions in biomarker levels after neoadjuvant therapy and prior to surgical resection (>90% reduction from baseline in CA19-9 levels and >50% reduction from baseline in CA19-9 levels (noted not to be a strict criterion), respectively). Additionally, Takeda et al. required that CA19-9 levels fall to normal ranges in response to neoadjuvant therapy before being selected for surgery. Four other studies stated that decreasing CA19-9 and/or CEA levels be noted prior to surgery but did not provide quantitative details regarding biomarker response [26,34,36,38]. The five remaining studies did not specify a biomarker response to neoadjuvant therapy that was required to undergo surgical resection [18,29,31,32,33].

Regarding radiologic response of the primary and metastatic lesions following neoadjuvant therapy, nine studies provided data [24,25,26,29,30,32,34,36,38]. Four studies characterized this response utilizing the RECIST (v1.1) criteria and mandated that patients have stable disease (SD), partial response (PR), or complete response (CR) following neoadjuvant treatment (CT/MRI/PET) [26,30,36]. Crippa et al. required that patients selected for surgery have complete radiologic response (RECIST v1.1) with zero to one lesion discoverable on pre-operative imaging modalities (CT/MRI/PET). Frigerio et al. stated that patients were only selected for surgery if a complete radiologic regression was observed following neoadjuvant therapies. Three studies detailed that a favorable radiologic response following neoadjuvant treatment was required for surgical candidacy but did not provide further specific details [24,29,32]. The remainder of the studies did not detail a specific neoadjuvant radiologic response that was utilized as a surgical selection criterion.

Surgical discretion played an essential role in all of the studies. In Hank et al., the surgical goal was to achieve R0 resection based on the discretion of the surgeon during intra-operative assessments, particularly concerning atypical liver resection. Crippa et al. similarly required intra-operative ultrasound to rule out occult disease or progression before proceeding with surgery, aiming for an R0 resection. Takeda et al., Frigerio et al., and Bachellier et al. all had the same objective of achieving R0 resection, and decisions were made intra-operatively based on factors like the patient’s response to therapy. Safi et al. and Yang et al. both included R0 resection as their surgical goal but did not provide further details about the specific intra-operative discretion criteria. In Hong et al., IRE was employed for cases where traditional R0 resection was not feasible due to the involvement of critical vascular structures, such as the portal vein or hepatic veins. Surgical discretion was exercised to determine the use of IRE versus resection based on tumor location, technical feasibility, and patient response to systemic chemotherapy. The primary surgical objective remained R0 resection or achieving local control with IRE in technically challenging cases. In the remaining studies, the surgical objective was similar, targeting R0 resection, but no details about the surgeon’s discretion were explicitly discussed.

Performance status, as quantified by the American Society of Anesthesiologists score (ASA) and/or the Eastern Cooperative Oncology Group score (ECOG), was utilized by five studies [25,29,30,33,36]. Takeda et al. and Shi et al. both required that patients have an ASA score no greater than three. One study, Yang et al., detailed that an ASA score no greater than two was required to undergo surgery. Two studies, Crippa et al. and Takeda et al. required that operative patients have an ECOG status no greater than two and one, respectively. Additionally, Frigerio et al. and Bachellier et al. required that pre-operative patients have a good performance status but did not provide quantitative details or a widely used scoring system.

### 3.6. Surgical Selection Factors for Metachronous and Mixed-Cohort Metastasis Patients

One study, Pausch et al., consisted of data from the SEER database; thus, specific surgical selection criteria and exact number of metachronous/synchronous patients were not able to be determined. Two studies, Hackert et al. and Nagai et al., reported surgical selection factors for cohorts that consisted of mixed synchronous and metachronous metastasis patients. Regarding number of hepatic lesions, Hackert et al. reported that patients with less than four hepatic metastatic lesions were considered for surgery while Nagai et al. specified a cut-off of less than five. Regarding size of hepatic lesions, strict criteria were not reported in either study; however, Hackert et al. did report that a majority (63/85) of resection patients did not have a liver lesion greater than two centimeters. Biomarker levels and radiologic response to neoadjuvant were not reported as strict criteria in either study. Hackert et al. specified that a smaller portion of their cohort received neoadjuvant treatment; however, the exact amount was not reported. Nagai et al. reported that a portion (37/47 patients) of their cohort did receive neoadjuvant treatment. Regarding hepatic locale of metastatic lesions, neither study reported strict criteria. Both studies noted that a goal of R0 resection per surgeon discretion was utilized in each of the resection patients. Nagai et al. indicated that surgery was performed in patients with a good performance status but did not specify exact scoring criteria utilized.

One study, Lu et al., reported surgical selection data for purely metachronous metastasis patients who received RFA of hepatic metastasis followed by primary PDAC resection at a separate time point. This study specified that resection patients do not have greater than three hepatic lesions that remained less than three centimeters in size. Hepatic locale of metastatic lesions was not specified as a criterion. Biomarker response to neoadjuvant therapy was not specified as a surgical selection criterion; however, authors did report that metastasis and primary demonstrate SD, PR, or CR (RECIST v1.1) following neoadjuvant treatment.

### 3.7. Overall Survival

Table 5 outlines the overall survival of the resection cohorts compared to the control in each study. Eight studies [18,25,26,29,30,32,33,36,37] compared median OS for patients with synchronous resection of the primary PDAC and hepatic metastasectomy (total cohort = 589) with patients who did not undergo resection (total cohort = 525). In these studies, the average median survival for synchronous resection patients was 21.8 months compared to 8.4 months for patients treated with chemotherapy alone or palliatively. Hackert and colleagues reported a split cohort (synchronous resection: n = 62, metachronous resection: n = 23), in which the median OS (12.3 months) was reported without comparison to a control.

Takeda and colleagues reported the highest median OS at 54.6 months, however, half of the patients (n = 5) in this cohort did not undergo synchronous resection due to intra-operatively undetectable hepatic lesions. The highest median OS following synchronous resection in the entire cohort was observed in Hank and colleagues, who reported a 25.5-month median OS in patients (ypM0, n = 45) with complete pathological response of the primary PDAC and metastasis on post-operative evaluation of resected specimens. It should be noted that this was in contrast to the lower median OS of 10.7 months in patients (ypM1, n = 48) with residual active metastasis in the resected specimens. Hank and colleagues reported a cohort with primarily hepatic oligometastatic (n = 67/93, 72%) but did include peritoneal and lymph node metastatic patients in their calculations. Safi and colleagues similarly reported a higher median OS of 17.6 months in synchronous resection patients (M1surgR0, n = 17) who had negative surgical margins compared to 10.3 months in patients with positive surgical margins (M1surg, n = 35) according to the R-classification system [39]. Three studies [31,32,36] reported a lower median OS for patients (n = 132, avg. median OS = 14.9 months) undergoing synchronous resection compared to controls (n = 319, avg. median OS = 22.5 months) who underwent only pancreatectomy for non-oligometastatic disease. Lu and colleagues reported favorable survival for patients defined as SD following pre-operative chemotherapy (RECIST v 1.1 criteria) who underwent RFA of liver metastases followed by pancreatectomy (n = 15, median OS = 16.8 months) compared with patients who underwent chemotherapy alone (n = 13, median OS = 12.9 months). Frigerio and colleagues reported only a median survival for patients with initial hepatic metastasis who had complete radiologic regression of metastases (18FDG-PET) and underwent resection of the primary PDAC only (n = 52, median OS = 23 months).

Three studies [25,26,30] evaluating primarily synchronous resection, which mandated either partial or stable response of PDAC to pre-operative chemotherapy based on the RECIST criteria (v 1.1) and a reduction in the tumor marker CA19-9 (Table 4), documented a superior median overall survival (n = 114, average median OS = 32.5 months) in contrast to patients (n = 148, average median OS = 9.2 months) who did not meet the RECIST criteria (v 1.1) and were treated palliatively. It should be noted that Crippa and colleagues observed complete regression of hepatic metastasis following primary chemotherapy in the majority of the cohort (n = 8/11, 72%) with only three patients requiring synchronous resection. Additionally, Hong et al. reported a median survival of 16 months (range 3–41) for a mixed cohort of patients who received neoadjuvant therapy followed by IRE in situ of the primary PDAC with either resection of external beam radiation of the oligometastasis.

### 3.8. Meta-Analysis of Overall Survival in Synchronous Resection and Metastasis Patients

**Figure 2:**
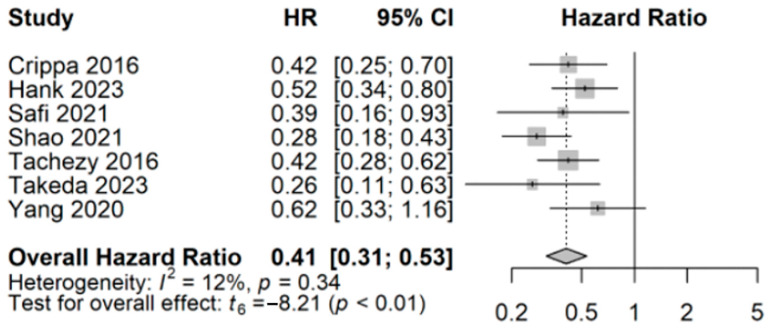
Comparative overall survival using pooled hazard ratios for surgically treated and non-surgically treated oligometastatic PDAC [25,26,29,30,31,32,33].

Seven studies reported univariate hazard ratios (HRs) that were pooled for meta-analysis [25,26,29,30,31,32,33]. All included studies reported an HR comparing the OS of synchronous resection and synchronous metastatic patients with a control cohort, which did not undergo resection of the primary or metastatic cancer. Meta-analysis estimated a pooled HR for OS to be 0.41 with a 95% confidence interval of 0.31 to 0.53 (Figure 2). This combined analysis indicates a statistically improved survival (*p* < 0.01) for patients who received synchronous resection compared to palliation. Heterogeneity was relatively low, as measured by I^2^ = 12%. Due to the differences in study design, inclusion criteria differed across studies included in the meta-analysis. Four studies [29,31,32,33] reported HRs of overall survival for strictly patients who underwent synchronous resection versus non-resection patients. Two studies reported a split cohort, in which either primary chemotherapy resulted in complete regression of hepatic metastasis (Crippa et al., n = 3/11) in a subset of patients or hepatic lesions were unable to be detected with ultrasound intra-operatively and were subsequently not resected (Takeda et al., n = 5/10). Hank et al. reported an HR that contained patients who had resection of peritoneal or lymph node metastasis (n = 26/93) in addition to synchronous hepatic resection patients (n = 67/93). The majority of the combined resection cohort consisted of patients undergoing synchronous resection of the primary PDAC and hepatic metastases (n = 269/308, 87%).

#### 3.8.1. Disease-Free Survival

Five studies reported median months (mo) of disease-free survival (DFS) [24,30,31,34,35]. Safi et al. reported a higher median DFS for the M1surgR0 (synchronous resection, negative margins) cohort (n = 17, median DFS (mo): 10.3, 95% CI: 3.3–17.4) compared to the M1surgR1 (synchronous resection, positive margins) cohort (n = 35, median DFS (mo): 4.4, 95% CI: 2.3–6.4) (*p* = 0.009). Additionally, the median DFS was significantly higher in the cohort that underwent only pancreatectomy for localized disease (n = 90, median DFS (mo): 12.9, 95% CI: 6.2–19.8) when compared to both M1surgR0 (*p* = 0.031) and M1surgR1 (*p* = 0.001). The highest median DFS was reported by Frigerio et al. (median DFS: 16.5 mo), in which the cohort received only pancreatectomy following complete radiologic regression of hepatic metastasis, as determined by a negative ^8^FDG-PET and CT/MRI. Takeda et al. reported the next highest median DFS (14 mo) in ten patients who underwent synchronous resection (n = 5/10) and pancreatectomy alone (n = 5/10). Bachellier et al. reported a median DFS of 5.4 months (95% CI: 4.5–6.9) in patients who underwent synchronous resection (n = 92). Nagai et al. reported a median DFS (6.1) for patients undergoing synchronous (n = 37/47) or metachronous resection (n = 10/47). Hong et al. reported two patients who underwent oligometastasectomy and/or primary PDAC treatment with IRE in situ who were still living with no evidence of recurrent disease at the date of last follow-up (DFS: 41 and 16 months, respectively). Regarding the remaining five patients, there were four with disease-specific mortality (range 3–29 months) and one who died from non-disease-related causes (30 months).

#### 3.8.2. Prognostic Indicators of Overall Survival

Table 6 provides a characterization of the prognostic factors for OS for patients undergoing resection. Seven studies reported prognostic factors for OS that were able to be extracted [24,26,27,29,33,34,35]. Regarding tumor markers, Bachellier et al. (synchronous resection and metastasis) reported that lower pre-operative CA19-9 levels (<500 kU/L) were associated with better survival on multivariate analysis (*p* = 0.003) similar to Nagai et al. (mixed synchronous/metachronous resection and metastasis) who reported that lower pre-operative CA19-9 levels (<200 kU/L) were associated with better survival on univariate analysis (*p* = 0.011) but not on multivariate analysis (*p* = 0.430). Bachellier et al. reported a benefit to survival in patients with a negative margin status on the resected primary tumor (multivariate, *p* = 0.020) similar to Nagai et al. who found negative margin status to be significant on univariate analysis (*p* = 0.011) but not multivariate analysis (*p* = 0.428). On univariate analysis, Tachezy et al. (synchronous resection and metastasis) found pancreatic head resection (*p* < 0.001) but not tail resection (*p* = 0.198) to be associated with a survival benefit compared to systemic chemotherapy alone. In contrast, Yang et al. (synchronous metastasis and resection) reported a survival benefit in patients with oligometastatic PDAC who underwent pancreatic body or tail resection (Kaplan–Meier, *p* = 0.0004) but not head resection (Kaplan–Meier, *p* = 0.74) when compared to non-oligometastatic (>3 hepatic lesion) resection patients. Regarding the role of chemotherapy, Nagai et al. found a negative survival benefit in resection patients who did not receive pre-operative chemotherapy (multivariate, *p* = 0.002) while Bachellier et al. (synchronous resection and metastasis) reported increased survival in resection patients receiving adjuvant chemotherapy (multivariate, *p* = 0.024). Regarding tumor differentiation, Nagai et al. found a survival benefit in resected patients whose primary tumor was moderate-to-well-differentiated (grades I–II) compared to poor (grade > II) differentiation (multivariate, *p* = 0.0003).

Of note, Hank et al. (synchronous resection and metastasis) reported a survival benefit in patients with lower pre-operative CA19-9 (<400 kU/L) (multivariate, *p* < 0.001), patients with complete pathologic responses of metastasis (multivariate, *p* = 0.011), and in patients who received adjuvant chemotherapy (multivariate, *p* = 0.003). Notably, Hank et al. did not report a difference in survival based on pre-operative chemotherapy regimens (FFX vs. GEM/GEM + FFX/other). Hackert et al. (mixed synchronous/metachronous resection and metastasis) did not identify any factors as having a benefit to survival including pre-operative CA19-9 levels and primary tumor location.

One study, Frigerio et al., reported prognostic factors for survival in a cohort of patients with initial hepatic oligometastatic PDAC that had complete response of metastases to pre-operative chemotherapy and underwent resection of the primary tumor only (Table 6). Of note, it was found that decreased pre-neoadjuvant chemotherapy CA19-9 and post-chemotherapy CA19-9 normalization were not associated with increased survival on univariate analysis (*p* = 0.830 and *p* = 0.437, respectively). On multivariate analysis, neutrophil-to-lymphocyte ratio (NLR < 1.7) and the Prognostic Nutritional Index (PNI > 53) were reported to be statistically associated with increased survival past twenty-four months (*p* = 0.019 and *p* = 0.008, respectively).

## 4. Discussion

This review is one of the first in recent years to assess the outcomes of surgical resection in hepatic metastatic PDAC patients. Our meta-analysis results indicate that there is increased OS in patients with synchronous hepatic metastasis who undergo synchronous resection. A recent phase II randomized trial (EXTEND) provided further evidence supporting the existence of an oligometastatic state in PDAC by offering insights into the role of metastasis-directed therapy (MDT) alongside standard systemic therapy [39]. The trial demonstrated a significant improvement in progression-free survival (PFS), with 10.3 months observed in the MDT group compared to 2.5 months in the control group. Enhanced systemic immune activation was noted as a potential mechanism contributing to this benefit. Importantly, no grade III or higher MDT-related adverse events were reported, which reinforces the safety and feasibility of integrating surgery with systemic treatment in stage IV PDAC patients.

In a previous meta-analysis, Yu et al. [40] found an increased OS in oligometastatic patients who underwent resection in addition to systemic therapy. This analysis, however, is limited by the fact that it includes a mixed cohort of synchronous and metachronous resection patients, which introduces substantial heterogeneity. A recent study on metastatic esophageal cancer suggested that there is a major difference in behavior regarding synchronous versus metachronous metastasis patients, with synchronous disease having better survival outcomes when compared with metachronous disease [41]. The current meta-analysis improves upon Lu and colleague’s findings by limiting the statistical analysis to only synchronous metastasis patients, which presents a more translucent perspective regarding the role of surgery in this patient population. Furthermore, the current study benefits from more modern chemotherapy regimens, such as FFX and nab-paclitaxel, which may have not been widely used in the older studies reviewed by Yu et al. Another recent review and critical synthesis on oligometastasis in PDAC patients performed by Leonhardt et al. reported a weighted median OS of 7.5 months (95% CI: 7.5–10.4) in those that underwent surgery. The current meta-analysis improves upon this synthesis by including studies with control groups, enabling the computation of pooled hazard ratios to provide a more robust assessment of overall survival. Additionally, while Leonhardt’s review included studies involving pulmonary metastases, our meta-analysis focuses exclusively on synchronous hepatic metastases, with only a small portion of peritoneal metastases included in the statistical analysis. Leonhardt et al. also highlighted that the majority of studies had a high risk of bias and often lacked control groups, which limits the reliability of their findings. Our narrower focus enhances the specificity of our findings and reflects the strong survival benefit in appropriately selected patients with hepatic-only synchronous oligometastasis who undergo synchronous surgical resection.

Additionally, we found that a number of potential selection factors are being used to define hepatic oligometastatic PDAC. There was a great degree of heterogeneity among statistical reporting, which prevented two of the key outcomes (surgical selection criteria and prognostic indicators of survival) from being included in the meta-analysis. In the qualitative analysis, there was a great degree of heterogeneity among surgical selection criteria between studies, however, the majority of studies specified that patients receive neoadjuvant therapy (FFX- or GEM-based) and have metastasis confined to the liver prior to surgery. The number of liver lesions considered to be oligometastatic varied widely across studies. While some studies included patients with up to three or four lesions, others did not specify limits. Most notably, there were five studies that did use less than four hepatic lesions as a criterion for surgical selection. The lack of a common definition of oligometastatic PDAC was highlighted by a recent systematic review performed by Leonhardt and colleagues [14]. This review found a wide range of hepatic lesions considered fit for surgery with some studies not utilizing this criterion at all. This likely reflects the complex nature of metastasis surgery, as when the type of liver resection was reported, most studies indicated a majority of metastases were resected atypically. Indeed, the results of this review may suggest that using the maximum number of liver lesions as a criterion for surgical selection is too arbitrary for this patient population. Instead, surgeons should potentially base patient selection on the ability to achieve R0 resection in the liver. For example, if the criterion is that patients receive surgery only if they have fewer than four hepatic lesions, but there are patients with five or six lesions that are tightly grouped, sub-capsular, and easily resectable, many patients who could benefit from surgery would be left out. Additionally, factors such as size of metastatic lesion and liver locale may play a greater role in surgical selection, however, this review was not able to identify a strict consensus in the studies reviewed. This lack of consistency in the literature regarding metastatic site and size was also reported in the review performed by Leonhardt and colleagues.

The use of RECIST v1.1 (Response Evaluation Criteria in Solid Tumors) to evaluate radiologic response is a potentially valuable tool in surgical selection The aforementioned measure is an objective assessment of tumor status that is easily evaluated on pre-operative imaging. A number of studies in this review utilized response to neoadjuvant therapy as graded by the RECIST v1.1 as a surgical selection factor. This review found some studies requiring that patients have SD, PR, or CR of hepatic metastasis and the primary PDAC following neoadjuvant therapy. However, there was no definite consensus in the studies reviewed with many only indicating that a favorable radiologic response be observed prior to proceeding with surgery. Notably, Lu et al. demonstrated a good median survival of 17 months in patients with at least stable disease (RECIST v1.1) after pre-operative chemotherapy who underwent radiofrequency ablation (RFA) of hepatic lesions followed by primary resection. Two recent studies found the RECIST classification to be associated with increased survival in patients treated with trans-arterial chemoembolization for hepatocellular carcinoma [42] and for ovarian cancer patients who undergo neoadjuvant therapy and tumor debulking [43]. The main limitation of studies using RECIST v1.1 as a criterion for surgical selection, both in this review and in the two aforementioned studies, remains the heterogeneity in the specific RECIST classifications considered appropriate for surgery by each individual surgeon. In agreement with Leonhardt and colleagues, a wide array of cut-offs were used from study to study regarding the utilization of biomarker response to neoadjuvant therapy as a surgical selection factor. The current literature demonstrates that lower CA19-9 levels can predict better survival in patients with PDAC who undergo non-operative management alone [44]. Similarly, several studies in this review found lower pre-operative CA19-9 to be predictive of increased post-operative survival. Standardizing biomarker response to neoadjuvant therapy is crucial for guiding surgeons in patient selection. However, based on the studies reviewed, there is no clear quantification beyond reductions in CA19-9 and CEA levels.

Performance status is a surgical selection factor that was unequivocally considered for each patient who underwent surgery. Some studies reported a quantification of this status by utilizing strict ASA or ECOG cut-offs. However, the majority of studies reviewed did not provide a consensus on hard cut-offs or the optimal performance status scoring system for hepatic oligometastatic PDAC patients. Most studies specified that a good performance status was required but without specific quantification.

In addition to the findings reported by Lu et al., the study by Hong et al. demonstrated the potential of combining a minimally invasive technique, IRE (versus RFA of hepatic lesions in Lu et al.) with traditional surgery and systemic chemotherapy to achieve promising outcomes in highly selected patients with stage IV PDAC. In agreement with Lu et al., patients in Hong et al. had to have a favorable response to neoadjuvant treatment per the RECIST criteria (stable or responsive) to be considered for surgery. The study reported a median overall survival (OS) of 16 months for the cohort, with two patients achieving profoundly prolonged survival and no evidence of recurrence. These studies highlight the need for further implementation and optimization of patient selection criteria for minimally invasive approaches in patients who have favorable tumor biology and a response to systemic chemotherapy. Incorporating such techniques into multidisciplinary treatment strategies could help increase surgical eligibility and improve outcomes in this challenging patient population.

This review highlights several key prognostic indicators of overall survival (OS) for patients undergoing resection of stage IV PDAC with hepatic metastases. A lack of standard reporting and various use of cut-offs prevented a meta-analysis from being performed. Nonetheless, across multiple studies, lower pre-operative CA19-9 levels consistently emerged as a significant predictor of improved survival outcomes. For example, Bachellier et al. and Nagai et al. both reported that patients with lower pre-operative CA19-9 levels following neoadjuvant therapy had a survival advantage, although only Bachellier’s findings were significant in multivariate analysis. This comparison however is complicated by the fact that the two aforementioned studies employ different CA19-9 cut-offs in their analyses (<500 μ/L vs. <200 μ/L, respectively). Similarly, achieving R0 resection status in both the primary tumor and metastatic sites was identified as a critical factor for survival, with several studies reporting a statistical association between negative margin status and improved OS. In terms of chemotherapy, receiving adjuvant chemotherapy post-resection was associated with better survival outcomes. Hank et al. also found that patients with a complete pathological response of metastases showed significantly improved survival compared to those with residual active disease, further emphasizing the importance of neoadjuvant therapy. These findings underscore the need for careful selection of patients for surgical intervention based on these prognostic factors, as they likely significantly influence post-operative OS.

The strengths of this review are related to its comprehensive nature and sound statistical methodology when applicable; however, there are limitations. The meta-analysis did include some data from patients who had only a pancreatectomy or who had peritoneal without hepatic metastasis. However, the majority of data in these studies included hepatic oligometastatic disease. If these studies that included peritoneal metastasis and pancreatectomy only were excluded, there would be insufficient data to complete a meta-analysis. Future studies should strive to separate their survival analyses in a stricter manner. The retrospective nature of the included studies introduces potential biases. Surgeon discretion and institutional expertise are significant confounding factors that are difficult to measure. High-volume hospitals with experienced surgeons are more likely to undertake complex resections, which allows for an unmeasurable bias regarding survival rates [45]. Heterogeneity in study design, control groups, resection cohorts, and clinicopathological characteristics limit the conclusions from statistical analysis. Additionally, there was a lack of subgroup analyses regarding the type of index operation performed, which does not allow for conclusions to be drawn pertaining to survival differences between various types of pancreatectomies. For instance, one study reported a survival benefit for head resections but not for tail resections, while another found the opposite. As more studies become available, differentiating whether distal pancreatectomy (DP) or pancreaticoduodenectomy (PD) confers better outcomes in stage IV PDAC is something we would like to investigate in the future.

Future research should strive for greater homogeneity in study designs and control groups to allow for more sound statistical analyses to be performed. The lack of a consistent definition of hepatic oligometastasis further complicates comparisons and underscores the need for a standardized classification system. Reaching a consensus on surgical selection criteria is critical for the continued research on surgery in oligometastatic PDAC patients. Incorporating indices like the Prognostic Nutritional Index (PNI) could aid in surgical selection as it allows an objective assessment of pre-operative nutritional status. In colorectal cancer patients, studies have demonstrated that a lower PNI is associated with increased post-operative complications, longer hospital stay, and decreased overall survival [46]. Identifying improved measures of performance status and pre-operative predictors of surgical success is something we will continue to investigate in the future. Additionally, there is increasing evidence that circulating tumor DNA (ctDNA), a form of liquid biopsy, is a useful prognostic measure for predicting OS and DFS. One recent study found lower ctDNA presence to be associated with lower OS and DFS in patients with locally resectable PDAC [47]. Although many institutions may not have retrospective ctDNA data available, it will likely play a significant role in future prognostication and surgical decision-making, and it is something we intend to investigate further. Additionally, separate survival analyses for patients receiving adjuvant/neoadjuvant chemotherapy versus those who do not would provide more granular insights regarding the role of surgery in this patient population. Overall, implementing a standardized framework for investigating the surgical treatment of these patients will enable more meaningful conclusions to be drawn.

## 5. Conclusions

This review highlights that the current literature supports a survival benefit in synchronous metastatic patient groups who undergo synchronous surgical resection of hepatic oligometastasis. Currently, there is no definite consensus regarding surgical selection factors in this patient population. However, the ability to achieve R0 resection and a favorable neoadjuvant response, both in terms of biomarker levels and radiologic findings, was identified as important, though the exact criteria varied between studies. Increased levels of homogeneity in statistical reporting and a common definition of hepatic oligometastasis will allow for more definitive conclusions.

## Figures and Tables

**Table 1:** Study characteristics.

Reference	Year	Inclusion Period	Country	Study Type	Number of Resected Patients	MINORS Grading
Pausch et al. [37]	2021	2010–2015	United States	Retrospective (SEER database)	259	20
Hank et al. [26]	2023	2006–2019	Germany	Retrospective	93	18
Hackert et al. [27]	2017	2001–2014	Germany	Retrospective	85	15
Hamad et al. [18]	2022	2010–2015	United States	Retrospective (NCB)	137	17
Crippa et al. [25]	2016	2003–2013	Italy	Retrospective	11	18
Lu et al. [28]	2023	2017–2020	China	Retrospective	15	14
Tachezy et al. [29]	2016	1994–2014	Europe	Retrospective	69	18
Takeda et al. [30]	2023	2013–2020	Japan	Retrospective	10	17
Safi et al. [31]	2021	2006–2019	Germany	Retrospective	35	18
Shao et al. [32]	2021	2009–2018	China	Retrospective	50	20
Yang et al. [33]	2020	2012–2017	China	Retrospective	23	18
Frigerio et al. [34]	2022	2008–2020	Italy and Austria	Retrospective	52	13
Bachellier et al. [24]	2022	2008–2020	France	Retrospective	92	14
Nagai et al. [35]	2023	2000–2019	United States	Retrospective	47	15
Shi et al. [36]	2016	2007–2015	China	Retrospective	30	14
Hong et al. [38]	2018	2010–2016	United States	Retrospective	7	17

The Surveillance, Epidemiology, and End Results (SEER) Program, National Cancer Database (NCB).

**Table 2:** Characterization of surgical procedures and their associated morbidity and mortality.

Author, Year	Patients with Resection of Stage IV PDAC	Timing of Metastasis Surgery	Surgery Type	30 d Mortality	90 d Mortality	Complications	Limitation
Pausch, 2021 [37]	259	NR	Author assumed curative-intent resection of all macroscopically evident sites of cancer	-	-	-	SEER database limitations
Hank, 2023 [26]	93 (n = 67/93 with only liver metastasis, n = 26/93 with peritoneal or lymph node resection)	Synchronous	PDAC resection: PD: 41/93 (44.1%) DP: 37/93 (39.8%) TP: 15/93 (16.1%) Liver resection 67/93 (72%): Atypical liver resection 58/67 (87.5%) Segmental resection 7/67 (9.7%) Hemi-hepatectomy 2/67 (2.8%)	None	3/92 3.2%.	Major complications (CD grades III–IV): 18/93 (19.4%)	
Hackert, 2017 [27]	85	62 (73%) synchronous 23 (27%) metachronous	PDAC resection: PD: 36 (42.4%) TP: 14 (16.5%) DP: 34 (40%) Liver resection 85/85 (100%): 1× atypical: 55/85 (64.7%) 2× atypical: 15/85 (17.7%) 3× atypical: 1/85 (1.1%) 4× atypical: 2/85 (2.4%) Bisegmentectomy: 2/85 (2.4%) Bisegmentectomy and 1× atypical: 3/85 (3.5%) R hepatectomy: 6/85 (7.1%) Extended R hepatectomy: 1/85 (1.1%)	1/62 (1.6%) (synchronous resection) 1/23 (4.3%) (metachronous resection)	-	30/62 (48%) (synchronous resections): Wound infection: 9/62 (14.5%) POPF: 6/62 (9.7%) DGE: 6/62 (9.7%) Lymphatic fistula: 5/62 (8.1%) Bleeding: 4/62 (6.4%) 5/23 (21.7%) (metachronous resection): Wound infection: 2/23 (8.6%) Bleeding: 1/23 (4.3%) Bilioma: 2/23 (8.6%)	All deaths included from any cause
Hamad, 2022 [18]	137	Synchronous	Primary and metastatic tumor resection	-	-		The National Cancer Database limitations
Crippa, 2016 [25]	11	8/11 no identifiable liver metastases at re-staging 3/11 synchronous	PDAC resection: PD: 6 (55%) DP: 5 (45%) Liver resection 3/11 (27.3%): Segmentectomy: 2/3 (66%) Atypical resection: 1/3 (33%)	-	-	Grade A pancreatic fistula (n = 2), post-operative pneumonia (n = 1)	
Lu, 2023 [28]	15	Metachronous (RFA followed by PDAC resection)	PDAC resection: PD: 9/15 (60%) DP: 6/15 (40%) RFA of liver metastasis 15/15 (100%)	-	-	-	
Tachezy, 2016 [29]	69	Synchronous	PDAC resection: PD: 30/69 (43.5%) PPPD: 12/69 (17.4%) TP: 2/69 (2.9%) DSP: 25/69 (36.2%) Liver resection 69/69 (100%): Type of resection not recorded	1/69 (1.4%)	-	Major complications (CD grades III–IV): 3/10 (30%)	
Takeda, 2023 [30]	10	5/10 synchronous resection 5/10 liver metastases unable to be detected intra-operatively	PDAC resection: SSPPD: 6/10 (60%) DP-CAR: 1/10 (10%) DP: 2/10 (20%) TP-CAR: 1/10 (10%) Liver resection: 5/10 (50%)	None	-	-	
Safi, 2021 [31]	35	Synchronous	-	-	-	-	
Shao, 2021 [32]	50	Synchronous	PDAC resection: PD: 50/50 (100%) Liver resection 50/50 (100%): Hepatic resection only: 45/50 (90%) Hepatic resection + RFA: 5/50 (10%)	-	-	POPF: 9/50 (18%) Hemorrhage: 4/50 (8%) DGE: 6/50 (12%) Infection: 5/50 (10%) Unplanned relaparotomy: 1/50 (2%)	
Yang, 2020 [33]	23	Synchronous	PDAC resection: PD: 12/23 (52%) DP: 11/23 (48%) Liver resection 23/23 (100%): Wedge resection: 21/23 (91%) Segmentectomy: 2/23 (9%)	None	-	Biochemical leak: 20/23 (87%) Grade B POPF: 3/23 (13%) DGE: 2/23 (9%) Biliary fistula: 3/23 (13%)	
Frigerio, 2022 [34]	52	None	PDAC resection: PD: 36/52 (69%) DP: 14/52 (27%) TP: 2/52 (4%)	None	-	POPF: 7/52 (13%) Hemorrhage: 3/52 (6%) DGE: 9/52 (17%) Other: 7/52 (13%) Overall morbidity = 57.7%	
Bachellier, 2022 [24]	92	Synchronous	PDAC resection: PD: 49/92 (53%) TP: 5/92 (5%) DSP: 38/92 (42%) Liver resection 92/92 (100%): Resection only: 62/92 (67%) RFA only: 19/92 (21%) Resection + RFA: 11/92 (12%)	-	5/92 (5%)	Major complications (CD grades III–IV): 14/92 (37%) POPF: 7/92 (7.6%)	
Nagai, 2023 [35]	47	37/47 synchronous 10/47 metachronous	PDAC resection: PD: 27/47 (57.4%) DP: 18/47 (38.3%) DP-CAR: 2/47 (4.3%) Liver resection 47/47 (100%)	None	-	Overall morbidity: 21/47 (45%) Major complications (CD grades III–IV): 8/47 37 (17%)	
Shi, 2016 [36]	30	Synchronous	PDAC resection: PD: 11/30 (36.7%) TP: 1/30 (3.3%) DP: 18/30 (60%) Liver resection 30/30 (100%)	-	-	POPF: 9/30 (30%) Chylous fistula: 1/30 (3.3%) DGE: 4/30 (13%) Infection: 6/30 (20%) Cerebral infarction: 1/30 (3.3%) Pneumonia: 1/30 (3.3%)	
Hong, 2018 [38]	7 (n = 4 with hepatic metastasis with or without omental/peritoneal metastasis, n = 3 with omental/peritoneal metastasis only)	2/7 synchronous 2/7 metastasis resection followed by IRE in situ 1/7 IRE in situ with metastasis resection 1/7 IRE in situ only 1/7 radiation therapy of metastasis followed by IRE in situ	PDAC resection: SP (½ with IRE margin accentuation): 2/7 (29%) IRE in situ (primary PDAC): 5/7 (71%) Liver resection 3/7 (43%): Segmentectomy: 2/3 (67%) Liver resection: 1/3 (33%) 3D conformal radiation therapy liver lesion x1: 1/7 (14%)	None	None	-	

Not recorded (NR), The Surveillance, Epidemiology, and End Results (SEER) Program, pancreaticoduodenectomy (PD), pancreatic ductal adenocarcinoma (PDAC), total pancreatectomy (TP), distal pancreatectomy (DP), subtotal pancreatectomy (SP), distal splenopancreatectomy (DSP), pylorus-preserving pancreaticoduodenectomy (PPPD), Clavien–Dindo complications (CD), delayed gastric emptying (DGE), post-operative pancreatic fistula (POPF), radiofrequency ablation (RFA), DP with celiac artery resection (DP-CAR), and irreversible electroporation (IRE).

**Table 3:** Chemotherapy regimens.

Author, Year	No. Patients with Resection of Stage IV PDAC	Neoadjuvant	Cycles	Adjuvant	Cycles
Hank, 2022 [26]	93	FFX (n = 61) GEM alone/other (n = 27) GEM + FFX (n = 5)	≥6, re-staging every 3 months to assess response	Individual basis based on pre-operative results	-
Hackert, 2017 [27]	85	NR	-	GEM (79.5%) 5-FU (8.2%) Other (12.3%).	-
Crippa, 2016 [25]	11	FFX or PEFG/PEXG/PDXG used on 77% of patients that achieved partial/complete response (only 11/45 of which went on to have surgery)	-	Only utilized after recurrence	-
Lu, 2023 [28]	15	FFX (n = 10) GnP (n = 5)	Chemo for 8–10 weeks, reassess stable/partial response, RFA for hepatic metastasis, chemo for 4–6 weeks, assessed as stable with resectable tumor	S-1 (n = 15)	Start 6–8 weeks after surgery
Tachezy, 2016 [29]	69	GEM (4% resected, 1% non-resected) FFX (6% resected, 0% non-resected)	-	GEM (65% resected, 70% non-resected) FFX (6% resected, 7% non-resected) (only includes 83% of the cohort’s data)	-
Takeda, 2023 [30]	10	GnP (n = 7) modified FFX (n = 2) S-IROX (n = 1)	-	S-1 (n = 10)	6 months
Safi, 2021 [31]	35	-	-	GEM (n = 15) FFX (n = 8) (four peri-operative and four post-operative) GEM + erlotinib or paclitaxel (n = 2) Radio-chemotherapy (n = 5)	-
Shao, 2021 [32]	50	FFX or GEM-based (n = 41)	-	76% chemo 10% chemo + RFA 8% chemo + radiotherapy	-
Yang, 2020 [33]	23	GnP (n = 2) RFA (n = 3)	-	79.2% (38/48) of synchronous resection patients received post- operative adjuvant chemotherapy GEM (7/38), GEM + oxaliplatin (13/38), GEM + S1 (11/38) and GnP (7/38)	-
Frigerio, 2022 [34]	52	FFX (n = 33) GnP (n = 14) GEM (n = 5)	Median: 9.4 (range 1–20)	-	-
Bachellier, 2022 [24]	92	FFX (n = 44) Other (n = 8)	Median: 9 (range 2–30)	n = 78/92	-
Nagai, 2023 [35]	47	FFX (n = 14) GnP (n = 4) FFX+ GnP (n = 9) Others (n = 5)	-	-	-
Hong, 2018 [38]	7	FFX (n = 2) FOLFOX (n = 1) PEXG (n = 1) GEM + 5-FU + radiation (n = 1) FFX + capecitabine (n = 1) GnP (n = 1)	Induction therapy followed by systemic therapy for 4–5 months	FOLFOX (n = 2) GEM (n = 1) Capecitabine, irinotecan (n = 1)	Based on individual treatment protocol

FOLFIRINOX (FFX), cisplatin, epirubicin, 5-fluorouracil, gemcitabine (PEFG), inguinal lymph node (ILN), cisplatin, epirubicin, capecitabine, gemcitabine (PEXG), cisplatin, capecitabine, docetaxel, gemcitabine (PDXG), gemcitabine (GEM), gemcitabine plus nab-paclitaxel (GnP), radiofrequency ablation (RFA), S-1, irinotecan and oxaliplatin (S-IROX), and 5-fluorouracil (5-FU).

**Table 4:** Surgical selection criteria for synchronous metastasis patients.

Study	Cohort Size	Neoadjuvant Therapy	Location	Number of Metastatic Lesions	Size	Liver Locale	Initial Biomarker Level	Neoadjuvant Response: Biomarker	Neoadjuvant Response: Radiologic	Surgeon Discretion	Performance Status
Hank, 2023 [26]	93 (67/93 hepatic-only, 26/93 peritoneal or lymphatic)	yes	Hepatic (with peritoneal and lymphatic)	NU	NU	NU (Atypical (58/67))	NU	Decreasing (CA19-9 and CEA)	Stable disease, partial response, or complete response (RECIST v1.1, CT/MRI/PET)	Goal of R0	NU
Hamad, 2022 (NCB limitations) [18]	137	yes (92/137—does not specify neoadjuvant vs. adjuvant)	Hepatic	NU	NU	NU	NU	NU	NU	Not specified	Not specified
Crippa, 2016 [25]	11	yes	Hepatic	No limit at diagnosis, 0–1 at re-staging	NU	NU	NU	>90% reduction from baseline (CA19-9)	Complete response (RECIST v1.1 CT/MRI/PET)	Goal R0, intra-operative U/S for r/o of occult disease/progression	ECOG status < 3
Tachezy, 2016 [29]	69	yes (10/69)	Hepatic	NU	NU	NU	NU	NU	Favorable response (non-specific)	Goal R0	ASA < 4
Takeda, 2023 [30]	10	yes	Hepatic	<4	NU	NU	NU	CA19-9 normalization	Stable disease, partial response, or complete response (RECIST v1.1, CT/MRI/PET)	Goal R0	ECOG status < 2
Safi, 2021 [31]	35	no	Hepatic	<5	NU	Confined to one lobe	NU	NU	NU	Goal R0	NU
Shao, 2021 [32]	50	yes (41/50)	Hepatic	NU	NU	NU	NU	NU	Favorable response (non-specific)	Goal R0	NU
Yang, 2020 [33]	23	yes (3/23)	Hepatic	<4	NU	NU	NU	NU	NU	Goal R0	ASA < 3
Frigerio, 2022 [34]	52	yes	Hepatic	0 (after re-staging)	NU	NU	NU	CA19-9 reduction (non-specific)	Complete radiologic regression	Goal R0	Good performance status (non-specific)
Bachellier, 2022 [24]	92	yes (52/92)	Hepatic	NU	<3 cm (after neoadjuvant therapy)	Sub-capsular	NU	>50% reduction from baseline (CA19-9) (not a strict criterion)	Favorable response (non-specific)	Goal R0	Good performance status (non-specific)
Shi, 2016 [36]	30	yes	Hepatic	NU	NU	NU	NU	Decreasing CA19-9 (non-specific)	Stable disease, partial response, or complete response (RECIST v1.1, CT/MRI/PET)	Goal R0	ASA < 4
Hong, 2018 [38]	7 (4/7 hepatic with or without omental/peritoneal metastasis)	yes	Hepatic (with peritoneal and omental)	<4	NU	No strict criterion mentioned	NU	Decreasing CA19-9 (non-specific)	Stable disease, partial response, or complete response (RECIST v1.1, CT/MRI/PET)	Goal R0 for traditionally resectable disease, IRE for unresectable/locally advanced tumors	NU

Not utilized (NU), Response Evaluation Criteria in Solid Tumors (RECIST), negative margin status resection (R0), Eastern Cooperative Oncology Group (ECOG), American Society of Anesthesiologists classification (ASA), and irreversible electroporation (IRE).

**Table 5:** Overall survival for patients with resection of stage IV PDAC versus palliative bypass/chemotherapy alone.

Study	Simultaneous Resections		Pancreatectomy with Liver RFA		Pancreatectomy Alone		Chemotherapy/Palliative/ Exploration		
	Median OS (95% CI) (mo)	n	Median OS (95% CI) (mo)	n	Median OS (95% CI) (mo)	n	Median OS (95% CI) (mo)	n	*p*-value
Pausch, 2021 [37]	range: (8–12)	259	NA	NA	NA	NA	range: (4–7)	259 (propensity score matched)	<0.001
Hank, 2022 [26]	ypM0: 25.5, ypM1: 10.7	ypM0: 45, ypM1: 48	NA	NA	NA	NA	8.1	80	<0.001
Hackert, 2017 [27]	12.3	85 *	NA	NA	NA	NA	NA	NA	NA
Hamad, 2022 [18]	15.6	137	NA	NA	NA	NA	8.1	137 (propensity score matched)	<0.001
Crippa, 2016 [25]	39	11	NA	NA	NA	NA	12	45	<0.0001
Lu, 2023 [28]	NA	NA	16.76 ± 6.55	15	NA	NA	12.92 ± 2.47	13	NR
Tachezy, 2016 [29]	14.5 (10.8–18.2)	69	NA	NA	NA	NA	7.5 (4.9–10.2)	69 (1:1 control)	<0.001
Takeda, 2023 [30]	54.6	10	NA	NA	NA	NA	20.8	23	<0.001
Safi, 2021—M1surg [31]	10.3 (7.2–13.4)	35	NA	NA	20.6 (16.7–24.6)	#	NR	NA	0.001
Safi, 2021—M1surg R0 [31]	17.6 (8.8–26.5)	17	NA	NA	20.6 (16.7–24.6)	#	NR	NA	NS
Shao, 2021 [32]	16	50	NA	NA	30	50 (1:1 control)	6	50 (1:1 control)	0.001
Yang, 2020 [33]	16.1	23	NA	NA	NA	NA	7.6 (chemotherapy), 4.3 (palliative)	31, 10	0.02, <0.0001
Frigerio, 2022 [34]	NA	NA	NA	NA	23	#	NA	NA	NA
Bachellier, 2022 [24]	12.7 (9.5–15.6)	92	NA	NA	NA	NA	NA	NA	NA
Nagai, 2023 [35]	12.3	47 *	NA	NA	NA	NA	NA	NA	NA
Shi, 2016 [36]	15.7	30	NA	NA	16.9	#	4.4	39	NR

* Includes both synchronous and metachronous resection patients, not available (NA), not recorded (NR), complete pathological response of resected primary PDAC and metastatic lesions (ypM0), residual metastasis in resected specimens (ypM1), negative margin status of resected specimens (R-classification) (M1surgR0), combined cohort with both positive and negative margins (R-classification) (M1surg), unspecified palliative treatment with or without chemotherapy (palliative*), and authors used statistical analysis to achieve 1:1 ratio of clinicopathological characteristics in resection vs. non-resection cohorts (1:1 control).

**Table 6:** Prognostic indicators of overall survival.

Author, Year	No. Patients with Resection of Stage IV PDAC	Prognostic Factor 1	Prognostic Factor 2	Prognostic Factor 3	Prognostic Factor 4	Prognostic Factor 5	Insignificant Factors
Hank, 2022 [26]	93 (MOTL)	Pre-operative CA19-9 ≥ 400 vs. < 400 U/mL (HR: 6.89, 95%CI: 2.96–16.03, *p* < 0.001) (MV)	ypM1 vs. ypM0 (HR: 1.99, 95% CI: 1.17–3.39, *p* = 0.011) (MV)	Adjuvant chemotherapy, yes vs. no (HR: 0.44, 95% CI: 0.26–0.75, *p* = 0.003) (MV)	-	-	ASA class III–IV vs. I–II (*p* = 0.075) (MV) Timing of operation (*p* = 0.289) (MV) Vascular involvement (*p* = 0.624) (MV) Neoadjuvant therapy: FFX vs. GEM/GF/O (*p* = 0.895) (MV)
Hackert, 2017 [27]	85 (MOTL)	-	-	-	-	-	Neoadjuvant therapy, yes or no (*p* = 0.287) No significant difference from the time of liver resection (*p* = 0.210) Pre-operative CA19-9 levels (*p* = 0.456) Number (one vs. two vs. three or more metastases; *p* = 0.589) Size of liver metastases (<1 cm vs. >1 cm; *p* = 0.713) Tumor location (head/body/tail) (*p* = 0.770).
Tachezy, 2016 [29]	69 (LOM)	No resection vs. resection (HR: 2.224, 95% CI: 1.448–3.415, *p* < 0.001) (MV)	Head resection (median OS 14.5 months) vs. no resection (median OS 7.0 months) *p* < 0.001) (K–M)	-	-	-	Age <65 vs. ≥65: (*p* = 0.194) (MV) Tail resection (median OS 14.0 months) vs. no resection (median OS 12.0 months) (*p* = 0.198) (K–M)
Yang, 2020 [33]	23 (LOM)	Body/tail resection: oligometastatic vs. non-oligometastatic 16.8 mo vs. 7.05 mo (*p* = 0.0004) (K–M)	-	-	-	-	Head resection: oligometastatic vs. non-oligometastatic: 6.9 mo vs. 5.5 mo (*p* = 0.74) (K–M)
Frigerio, 2022 [34]	52 (no hepatic resection)	OS > 24 mo: Operation time < 330 min (*p* = 0.032) (MV)	OS > 12 mo: No vascular resection (*p* = 0.005) (MV)	OS > 24 mo: NLR < 1.7 (*p* = 0.019) (MV)	OS > 24 mo: PNI > 53 (*p* = 0.008) (MV)	-	CA19-9 decrease and post-treatment normalization (UV) N-status (UV) Margin status (UV) Complete pathologic response (UV)
Bachellier, 2022 [24]	92 (LOM)	Pre-operative CA19-9 levels <500 kU/L HR: 0.35; 0.17–0.70 (*p* = 0.003) (MV)	R0 resection HR: 0.46; 0.24–0.88 (*p* = 0.020) (MV)	Adjuvant chemotherapy HR: 0.39; 0.17–0.88 (*p* = 0.024) (MV)	-	-	-
Nagai, 2023 [35]	47 (LOM)	No pre-operative chemotherapy (HR = 7.1 (2.13–23.3 (*p* = 0.002) (MV)	Moderate–well differentiation of the primary tumor median OS: 36.9 vs. poor differentiation median OS: 17.7 months (HR = 3.7; *p* = 0.003) (MV)	Median OS: pre-operative CA19-9 ≤ 200 vs. CA19-9 > 200 (26.3 vs. 19.1 months, *p* = 0.043 (UV)	Primary tumor resection margin (R0 vs. R1) (*p* = 0.011) (UV)	-	Number of liver metastases (N ≤ 1 vs. N > 1) (*p* = 0.596 (UV) Extent of liver surgery (*p* = 0.927 (UV) Lymph node metastasis (*p* = 0.269) (UV) Response to pre-operative chemotherapy (G1/G2 vs. G3) (*p* = 0.320) (UV) Pre-operative CA19-9 vs. ≤ 200 vs. CA19-9 > 200 (*p* = 0.430) (MV) T factor (T0/T1/T2 vs. T3/T4) (*p* = 0.727) (MV) Primary tumor resection margin (R0 vs. R1) (*p* = 0.428) (MV)

American Society of Anesthesiologists classification (ASA), overall survival (OS), FOLFIRINOX (FFX), gemcitabine (GEM), FFX + GEM (GF), other (O), metastatic sites other than liver included in cohort (MOTL), liver-only metastasis (LOT), hazard ratio (HR), multivariate (MV), univariate (UV), Kaplan–Meier survival curve (K–M), patients with residual metastasis (ypM1), patients with complete pathological response of specimens (ypM0), Prognostic Nutritional Index (PNI), neutrophil-to-lymphocyte ration (NLR), negative margin status of resected specimens (R-classification) (R0), positive margin status of resected specimens (R-classification) (R1), well-differentiated tumor (G1), moderately differentiated tumor (G2), and poorly differentiated (G3) T factor (T0/T1/T2/T3/T4) (TNM staging system—size and extent of primary tumor).

## Data Availability

The data presented in this study are available in this article.

## References

[1] Azar I., Virk G., Esfandiarifard S., Wazir A., Mehdi S. (2019). Treatment and survival rates of stage IV pancreatic cancer at VA hospitals: A nation-wide study. J. Gastrointest. Oncol..

[2] Sung H., Ferlay J., Siegel R.L., Laversanne M., Soerjomataram I., Jemal A., Bray F. (2021). Global Cancer Statistics 2020: GLOBOCAN Estimates of Incidence and Mortality Worldwide for 36 Cancers in 185 Countries. CA Cancer J. Clin..

[3] Jiang Y., Abboud Y., Liang J., Larson B., Osipov A., Gong J., Hendifar A.E., Atkins K., Liu Q., Nissen N.N. (2024). The Disproportionate Rise in Pancreatic Cancer in Younger Women Is Due to a Rise in Adenocarcinoma and Not Neuroendocrine Tumors: A Nationwide Time-Trend Analysis Using 2001–2018 United States Cancer Statistics Databases. Cancers.

[4] Siegel R.L., Giaquinto A.N., Jemal A. (2024). Cancer statistics, 2024. CA Cancer J. Clin..

[5] Rahib L., Smith B.D., Aizenberg R., Rosenzweig A.B., Fleshman J.M., Matrisian L.M. (2014). Projecting cancer incidence and deaths to 2030: The unexpected burden of thyroid, liver, and pancreas cancers in the United States. Cancer Res..

[6] Mizrahi J.D., Surana R., Valle J.W., Shroff R.T. (2020). Pancreatic cancer. Lancet.

[7] Conroy T., Hammel P., Hebbar M., Ben Abdelghani M., Wei A.C., Raoul J.-L., Choné L., Francois E., Artru P., Biagi J.J. (2018). FOLFIRINOX or Gemcitabine as Adjuvant Therapy for Pancreatic Cancer. N. Engl. J. Med..

[8] Puckett Y., Garfield K. (2024). Pancreatic Cancer. StatPearls.

[9] Das S., Batra S. (2015). Pancreatic Cancer Metastasis: Are we being Pre-EMTed?. Curr. Pharm. Des..

[10] Kirkegard J., Gaber C., Heide-Jorgensen U., Fristrup C.W., Lund J.L., Cronin-Fenton D., Mortensen F.V. (2024). Effect of surgery versus chemotherapy in pancreatic cancer patients: A target trial emulation. J. Natl. Cancer Inst..

[11] Von Hoff D.D., Ervin T., Arena F.P., Chiorean E.G., Infante J., Moore M., Seay T., Tjulandin S.A., Ma W.W., Saleh M.N. (2013). Increased Survival in Pancreatic Cancer with nab-Paclitaxel plus Gemcitabine. N. Engl. J. Med..

[12] Burris H.A., Moore M.J., Andersen J., Green M.R., Rothenberg M.L., Modiano M.R., Cripps M.C., Portenoy R.K., Storniolo A.M., Tarassoff P. (2023). Improvements in Survival and Clinical Benefit With Gemcitabine as First-Line Therapy for Patients With Advanced Pancreas Cancer: A Randomized Trial. J. Clin. Oncol..

[13] Singh I., Chou J.F., Capanu M., Park J., Yu K.H., Varghese A.M., Park W., Zervoudakis A., Keane F., Rolston V.S. (2024). Morbidity and mortality in patients with stage IV pancreatic adenocarcinoma and acute cholangitis: Outcomes and risk prognostication. Pancreatology.

[14] Leonhardt C.S., Stamm T., Hank T., Prager G., Strobel O. (2023). Defining oligometastatic pancreatic cancer: A systematic review and critical synthesis of consensus. ESMO Open.

[15] Liu M., Wang M., Li S. (2021). Prognostic Factors of Survival in Pancreatic Cancer Metastasis to Liver at Different Ages of Diagnosis: A SEER Population-Based Cohort Study. Front. Big Data.

[16] Creasy J.M., Sadot E., Koerkamp B.G., Chou J.F., Gonen M., Kemeny N.E., Balachandran V.P., Kingham T.P., DeMatteo R.P., Allen P.J. (2018). Actual 10-year survival after hepatic resection of colorectal liver metastases: What factors preclude cure?. Surgery.

[17] Hellman S., Weichselbaum R.R. (1995). Oligometastases. J. Clin. Oncol..

[18] Hamad A., Underhill J., Ansari A., Thayaparan V., Cloyd J.M., Li Y., Pawlik T.M., Tsung A., Abushahin L., Ejaz A. (2022). Surgical treatment of hepatic oligometastatic pancreatic ductal adenocarcinoma: An analysis of the National Cancer Database. Surgery.

[19] Halle-Smith J.M., Powell-Brett S., Roberts K., Chatzizacharias N.A. (2023). Resection of isolated liver oligometastatic disease in pancreatic ductal adenocarcinoma: Is there a survival benefit? A systematic review. World J. Gastrointest. Surg..

[20] Liberati A., Altman D.G., Tetzlaff J., Mulrow C., Gotzsche P.C., Ioannidis J.P., Clarke M., Devereaux P.J., Kleijnen J., Moher D. (2009). The PRISMA statement for reporting systematic reviews and meta-analyses of studies that evaluate healthcare interventions: Explanation and elaboration. BMJ.

[21] Slim K., Nini E., Forestier D., Kwiatkowski F., Panis Y., Chipponi J. (2002). Methodological index for non-randomized studies (minors): Development and validation of a new instrument. ANZ J. Surg..

[22] Tierney J.F., Stewart L.A., Ghersi D., Burdett S., Sydes M.R. (2007). Practical methods for incorporating summary time-to-event data into meta-analysis. Trials.

[23] Hirst T.C., Sena E.S., Macleod M.R. (2021). Using median survival in meta-analysis of experimental time-to-event data. Syst. Rev..

[24] Bachellier P., Addeo P., Averous G., Dufour P. (2022). Resection of pancreatic adenocarcinomas with synchronous liver metastases: A retrospective study of prognostic factors for survival. Surgery.

[25] Crippa S., Bittoni A., Sebastiani E., Partelli S., Zanon S., Lanese A., Andrikou K., Muffatti F., Balzano G., Reni M. (2016). Is there a role for surgical resection in patients with pancreatic cancer with liver metastases responding to chemotherapy?. Eur. J. Surg. Oncol..

[26] Hank T., Klaiber U., Hinz U., Schutte D., Leonhardt C.S., Bergmann F., Hackert T., Jager D., Buchler M.W., Strobel O. (2023). Oncological Outcome of Conversion Surgery After Preoperative Chemotherapy for Metastatic Pancreatic Cancer. Ann. Surg..

[27] Hackert T., Niesen W., Hinz U., Tjaden C., Strobel O., Ulrich A., Michalski C.W., Buchler M.W. (2017). Radical surgery of oligometastatic pancreatic cancer. Eur. J. Surg. Oncol..

[28] Lu W., Wang L., Lou J., Tang K. (2023). Sequential therapy for pancreatic cancer patients with synchronous oligo-hepatic metastatic lesions. Tumori.

[29] Tachezy M., Gebauer F., Janot M., Uhl W., Zerbi A., Montorsi M., Perinel J., Adham M., Dervenis C., Agalianos C. (2016). Synchronous resections of hepatic oligometastatic pancreatic cancer: Disputing a principle in a time of safe pancreatic operations in a retrospective multicenter analysis. Surgery.

[30] Takeda T., Sasaki T., Okamoto T., Kasuga A., Matsuyama M., Ozaka M., Inoue Y., Takahashi Y., Saiura A., Sasahira N. (2023). Outcomes of pancreatic cancer with liver oligometastasis. J. Hepatobiliary Pancreat Sci..

[31] Safi S.A., Fluegen G., Rehders A., Haeberle L., Fung S., Keitel V., Krieg A., Knoefel W.T., Lehwald-Tywuschik N. (2021). Surgical margin clearance and extended chemotherapy defines survival for synchronous oligometastatic liver lesions of the ductal adenocarcinoma of the pancreas. Int. J. Clin. Oncol..

[32] Shao Y., Feng J., Hu Z., Wu J., Zhang M., Shen Y., Zheng S. (2021). Feasibility of pancreaticoduodenectomy with synchronous liver metastasectomy for oligometastatic pancreatic ductal adenocarcinoma—A case-control study. Ann. Med. Surg..

[33] Yang J., Zhang J., Lui W., Huo Y., Fu X., Yang M., Hua R., Wang L., Sun Y. (2020). Patients with hepatic oligometastatic pancreatic body/tail ductal adenocarcinoma may benefit from synchronous resection. HPB.

[34] Frigerio I., Malleo G., de Pastena M., Deiro G., Surci N., Scopelliti F., Esposito A., Regi P., Giardino A., Allegrini V. (2022). Prognostic Factors After Pancreatectomy for Pancreatic Cancer Initially Metastatic to the Liver. Ann. Surg. Oncol..

[35] Nagai M., Wright M.J., Ding D., Thompson E.D., Javed A.A., Weiss M.J., Hruban R.H., Yu J., Burkhart R.A., He J. (2023). Oncologic resection of pancreatic cancer with isolated liver metastasis: Favorable outcomes in select patients. J. Hepatobiliary Pancreat Sci..

[36] Shi H.J., Jin C., Fu D.L. (2016). Preoperative evaluation of pancreatic ductal adenocarcinoma with synchronous liver metastasis: Diagnosis and assessment of unresectability. World J. Gastroenterol..

[37] Pausch T.M., Liu X., Cui J., Wei J., Miao Y., Heger U., Probst P., Heap S., Hackert T. (2021). Survival Benefit of Resection Surgery for Pancreatic Ductal Adenocarcinoma with Liver Metastases: A Propensity Score-Matched SEER Database Analysis. Cancers.

[38] Hong Y., Rice J., Sharma D., Martin R.C.G. (2018). The use of IRE in multi-modality treatment for oligometastatic pancreatic cancer. Am. J. Surg..

[39] Kumar N.A.N., Palod A., Usman N., Ahmed S. (2024). Periarterial and Sub-adventitial Divestment Along with Triangle Operation and RAMPS for Pancreatic Body Cancer. Ann. Surg. Oncol..

[40] Yu X., Gu J., Fu D., Jin C. (2017). Dose surgical resection of hepatic metastases bring benefits to pancreatic ductal adenocarcinoma? A systematic review and meta-analysis. Int. J. Surg..

[41] Pape M., Vissers P.A.J., Bertwistle D., McDonald L., Slingerland M., Haj Mohammad N., Beerepoot L.V., Ruurda J.P., Nieuwenhuijzen G.A.P., Jeene P.M. (2022). A population-based study in synchronous versus metachronous metastatic esophagogastric adenocarcinoma. Ther. Adv. Med. Oncol..

[42] Shuster A., Huynh T.J., Rajan D.K., Marquez M.A., Grant D.R., Huynh D.C., Jaskolka J.D. (2013). Response Evaluation Criteria in Solid Tumors (RECIST) criteria are superior to European Association for Study of the Liver (EASL) criteria at 1 month follow-up for predicting long-term survival in patients treated with transarterial chemoembolization before liver transplantation for hepatocellular cancer. J. Vasc. Interv. Radiol..

[43] Bogani G., Matteucci L., Tamberi S., Ditto A., Sabatucci I., Murgia F., Arcangeli V., Maltese G., Comerci G., Stefanetti M. (2019). RECIST 1.1 criteria predict recurrence-free survival in advanced ovarian cancer submitted to neoadjuvant chemotherapy. Eur. J. Obs. Gynecol. Reprod. Biol..

[44] Yang G.Y., Malik N.K., Chandrasekhar R., Ma W.W., Flaherty L., Iyer R., Kuvshinoff B., Gibbs J., Wilding G., Warren G. (2013). Change in CA 19-9 levels after chemoradiotherapy predicts survival in patients with locally advanced unresectable pancreatic cancer. J. Gastrointest. Oncol..

[45] Mehta H.B., Parmar A.D., Adhikari D., Tamirisa N.P., Dimou F., Jupiter D., Riall T.S. (2016). Relative impact of surgeon and hospital volume on operative mortality and complications following pancreatic resection in Medicare patients. J. Surg. Res..

[46] Xie H., Wei L., Yuan G., Liu M., Tang S., Gan J. (2022). Prognostic Value of Prognostic Nutritional Index in Patients With Colorectal Cancer Undergoing Surgical Treatment. Front. Nutr..

[47] Lee B., Lipton L., Cohen J., Tie J., Javed A.A., Li L., Goldstein D., Burge M., Cooray P., Nagrial A. (2019). Circulating tumor DNA as a potential marker of adjuvant chemotherapy benefit following surgery for localized pancreatic cancer. Ann. Oncol..

